# Impact of Different Mineral Reinforcements on HDPE Composites: Effects of Melt Flow Index and Particle Size on Physical and Mechanical Properties

**DOI:** 10.3390/polym16142063

**Published:** 2024-07-19

**Authors:** Pedro Henrique Poubel Mendonça da Silveira, Marceli do Nascimento da Conceição, Davi Nascimento de Pina, Pedro Afonso de Moraes Paes, Sergio Neves Monteiro, Neyda de La Caridad Om Tapanes, Roberto Carlos da Conceição Ribeiro, Daniele Cruz Bastos

**Affiliations:** 1 Department of Materials Science, Military Institute of Engineering—IME, Praça General Tibúrcio 80, Urca, Rio de Janeiro 22290-270, RJ, Brazil; 2 Centre for Mineral Technology—CETEM, Rio de Janeiro 21941-908, RJ, Brazil; marceli@metalmat.ufrj.br (M.d.N.d.C.); rcarlos@cetem.gov.br (R.C.d.C.R.); 3 West Zone Campus, Rio de Janeiro State University—UERJ, Avenida Manuel Caldeira de Alvarenga, 1203, Campo Grande, Rio de Janeiro 23070-200, RJ, Brazil; davi.pina@hotmail.com (D.N.d.P.); pedromoraespaes@gmail.com (P.A.d.M.P.); snevesmonteiro@gmail.com (S.N.M.); neyda.tapanes@uerj.br (N.d.L.C.O.T.)

**Keywords:** HDPE, bahia beige, limestone, MFI, particle size, izod, tensile, density, SEM, FRX

## Abstract

The use of mineral reinforcements in polymer matrix composites has emerged as an alternative for sustainable production, reducing waste and enhancing the physical and mechanical properties of these materials. This study investigated the impact of the melt flow index (MFI) of HDPE and the particle size of two mineral reinforcements, Bahia Beige (BB) and Rio Grande do Norte Limestone (CRN), on the composites. All composites were processed via extrusion, followed by injection, with the addition of 30 wt.% reinforcement. Chemical analyses revealed similar compositions with high CaO content for both minerals, while X-ray diffraction (XRD) identified predominantly calcite, dolomite, and quartz phases. Variations in the MFI, reinforcement type, and particle size showed a minimal influence on composite properties, supported by robust statistical analyses that found no significant differences between groups. Morphological analysis indicated that composites with lower MFI exhibited less porous structures, whereas larger particles of BB and CRN formed clusters, affecting impact resistance, which was attributed to poor interfacial adhesion.

## 1. Introduction

Based on the quantity of waste produced, it is anticipated that the treatment and disposal of solid waste will generate 1.6 billion tons of carbon dioxide (CO_2_) emissions, equivalent to greenhouse gas (GHG) emissions, accounting for 5% of global emissions. This is particularly noticeable in contexts where there are still issues with inadequate waste management. Approximately 40% of global waste is disposed of in landfills, 19% is recovered through recycling and composting, and 19% is treated through modern incineration systems, although 33% of waste is still openly dumped worldwide. Waste disposal practices vary greatly, depending on the level and region. In low-income countries, approximately 93% of waste is burned or dumped in roads, open landfills, or watercourses, while only 2% of waste is improperly disposed of in higher-income nations [1].

The concept of a circular economy seeks to change traditional practices of production and economic growth, which were considered in the linear economy. Alongside the circular economy model, in 2015, the United Nations (UN) launched the 2030 Agenda for Sustainable Development [2,3]. The 2030 Agenda encompasses 17 Sustainable Development Goals (SDGs) and 169 targets that can be measured using 230 qualitative data and quantitative indicators. Related to waste production and management, SDG 12 (responsible consumption and production) sets a specific deadline of 2030 to reduce losses and waste generation through the implementation of prevention, recycling, and reuse measures [2]. Additionally, SDG 11 (Sustainable Cities and Communities) and SDG 14 (Life Below Water) are directly related to the soundness of waste management (i.e., sustainable waste management practices, marine pollution of plastics and microplastics, etc.) [4,5,6].

In addition to the aforementioned strategies, in 2019, the European Green Deal (EGD) was launched as a guideline aimed at making the EU’s economic system more sustainable, turning climate and environmental concerns into opportunities for all, with policy priorities. Its goals include, among others, (i) a 90% reduction in transport emissions, (ii) a GHG reduction target for 2030 of at least 50% and up to 55% compared to the 1990 levels, (iii) carbon-neutral steel by 2030, and (iv) endorsing a circular economy [1,7]. While sustainable development constitutes a key element of government agendas, the lack of individuals with sustainability expertise is a major risk. Thus, ensuring that educational institutions such as universities and research centers are the pillars of sustainable communities is of utmost importance [8,9,10].

In the mining sector, during the extraction of ores and minerals, residues such as extremely fine particles are rejected from the grinding, screening, or processing of raw materials and are typically disposed of in large impoundments [11]. The reuse of these secondary resources is highly encouraged due to the amount generated and the economic and environmental costs associated with their management [12,13]. It is possible to extract significant contents of raw (critical) minerals, especially from residues of old mines explored with obsolete technologies [14].

However, the negative environmental impacts caused by these residues are severe. They promote the loss and degradation of natural areas and harm the health of the population exposed to toxic metals resuspended as dust. Freshwater ecosystems are particularly degraded both because they receive sediment loads, including chemical compounds used in processing, and because they are impacted by tailing dam failures, which devastate thousands of kilometers of rivers and streams with millions of tons of mud and toxic waste [15,16,17,18]. Another underestimated impact is groundwater contamination, which occurs when rainwater infiltrates waste and transports large amounts of metals and other contaminants into groundwater bodies [19,20]. All of these problems raise social awareness to reduce environmental risk and liability, and they are particularly relevant for the countries with the largest mineral reserves, such as the United States, Australia, India, Russia, China, and Brazil.

Brazil is one of the world’s largest producers of natural rocks, and tied to this production is the generation of a large amount of waste. Thinking in a circular manner reveals that there is an opportunity to utilize the waste from the ornamental rock production chain in the manufacture of artificial materials, not only generating ecological products with good technological properties and market potential but also collaborating to mitigate the environmental problem of waste disposal. Brazilian exports of natural rock materials for ornamentation and cladding totaled US$ 987.4 million and 2.16 million tons in 2020, according to the Brazilian Association of Ornamental Stones Industry [21,22].

The use of mineral reinforcements in polymer composites allows for the reuse of mineral waste, which would otherwise have no utility for society and would be discarded in landfills [23,24,25]. An example of mineral waste originally used as ornamental stone is Bahia Beige marble. Also known in Brazil as Bege Bahia, this marble is a natural stone widely used due to its aesthetic appeal, strength, and versatility. Originating from the state of Bahia, Brazil, this sedimentary rock possesses unique characteristics that make it a popular choice for various architectural and decorative applications. With its soft tonality ranging from beige to cream and its elegant and homogeneous texture, Bahia Beige is extensively used in architecture and design, adding sophistication and style to residential and commercial environments [26]. The application of Bahia Beige in composites has been little studied, although there are some reports in the literature that address the use of this material as a reinforcing agent. Santos et al. [27] processed polypropylene composites with Bahia Beige fractions ranging from 10 to 50 wt.% and analyzed the physical, chemical, and mechanical properties of these composites. Due to processing via extrusion, followed by hot pressing, the authors observed good interactions between the mineral and the polymer matrix, as evidenced in FTIR, XRD, and SEM, resulting in increased hardness. However, due to the ceramic nature of the reinforcement, the material’s ductility was reduced, resulting in low impact resistance. Nonetheless, due to the higher thermal stability, the material performed better at higher temperature ranges, achieving superior results at temperatures of approximately 150 °C for additions up to 40 wt.% of Bahia Beige, indicating potential for applications involving high temperatures.

Another abundant mineral in Brazil is limestone, especially in the state of Rio Grande do Norte, which has a vast reserve of this resource. The region contains over 20,000 km^2^ of limestone rocks. Limestone is extremely important for the local population, as it is used in construction, agriculture, and soil acidity correction [28].Limestone is a sedimentary rock that is the main source of lime material, widely used in the construction industry and agriculture. Limestone is mainly composed of the minerals calcite and aragonite, which are different crystalline forms of calcium carbonate, CaCO_3_. Limestone mining for lime production is an important economic activity in the state of Rio Grande do Norte, which exports lime throughout Brazil [29]. Limestone has also been used as reinforcement in engineering composites. Vinayagamoorthy [30] produced hybrid composites with a polypropylene matrix, jute fabric at 10 wt.%, and limestone at 20 wt.%, varying the particle size from 0.2 to 1 mm. Mechanical tests including tensile, flexural, and compression tests showed that increasing the average particle size, combined with jute fabric, enhanced the mechanical strength. The group with an average particle size of 0.8 mm exhibited improved tensile and impact resistance, whereas the 0.6-mm average size resulted in a higher compression strength.

Polyethylene (PE) is one of the most widely used thermoplastics in the world due to its resistance, nearly zero moisture absorption, chemical inertness, strong dielectric character, low friction, and ease of processing [31,32]. Pipes, containers, electrical insulation, and various other items are made from PE [33,34]. The mechanical and physical properties of PE depend significantly on variables such as crystallinity content, structure, and molecular weight [35]. Composites made with PE as a matrix have good mechanical and physical properties compared to pure polymer, and they can be used as packaging materials, in electrical storage devices, and in thermal, automotive, ballistic, and aerospace applications. PE can be extruded, injected, or compression-molded [36,37,38,39,40,41,42].

When working with polymer composites, certain factors are crucial for obtaining high-quality materials. One of these factors is the melt flow index (MFI) of the matrix, as the properties of these materials are closely linked to the quality of the mixture [43], which is affected by the MFI of the polymer matrix. The MFI is a parameter used in polymers for raw material quality control. It is inversely proportional to viscosity and, therefore, depends on the molecular properties of the polymers. During the preparation of composites, the polymer’s fluidity can influence the selection of aggregates and the wetting process, contributing to a more uniform distribution [44].

During the processing of thermoplastic composites, the wetting stage of the reinforcements via the molten polymer promotes an interaction between the matrix and reinforcement phases. The structure and properties of the matrix/fiber interface influence the final properties of the composites [45]. Good interfacial adhesion increases the stress transmission from the matrix to the fiber, improving the mechanical performance of the composite. Thus, using a polymeric matrix with an adequate fluidity index for the reinforcement, along with appropriate processing parameters, can result in different compositional characteristics [46].

Based on the premise presented in this introduction, the objective of this paper is to evaluate the physical and mechanical properties of high-density polyethylene (HDPE) composites with two different melt flow indices, reinforced with Bahia Beige limestone and limestone from Rio Grande do Norte. To determine which melt flow index provides the best properties in relation to the type of reinforcement, tests for density, hardness, tensile strength, impact resistance, X-ray diffraction, and X-ray fluorescence were conducted. Additionally, a detailed statistical analysis was performed to comprehensively compare the physical and mechanical properties of the composites.

## 2. Materials and Methods

### 2.1. Raw Materials

In this study, high-density polyethylene (HDPE) from Braskem was used with two different melt flow indices (MFIs): 7 g/10 min and 25 g/10 min, according to the ASTM D-1238 standard [47]. The aim of this work was to compare the performance of reinforcements in HDPE, with both high and low MFIs, thus investigating the best configuration for application in composites. The rock waste was donated by the Center for Mineral Technology (CETEM), consisting of Bahia Beige (BB) marble waste (sieved with diameters of D_1_ < 20 μm (or 635 #) and 20 μm < D_2_ < 44 μm (or 325 #)) and limestone waste from Rio Grande do Norte (CRN) (diameters: D_1_ < 20 μm and 20 μm < D_2_ < 44 μm). Before the extrusion of the composites, both BB and CRN powders were sieved to obtain the desired particle size distribution. Subsequently, they were dried in an oven at 80 °C for 24 h to prevent moisture’s presence and avoid interference with composite interfacial adhesion.

### 2.2. Composite Processing

**Figure 1:**
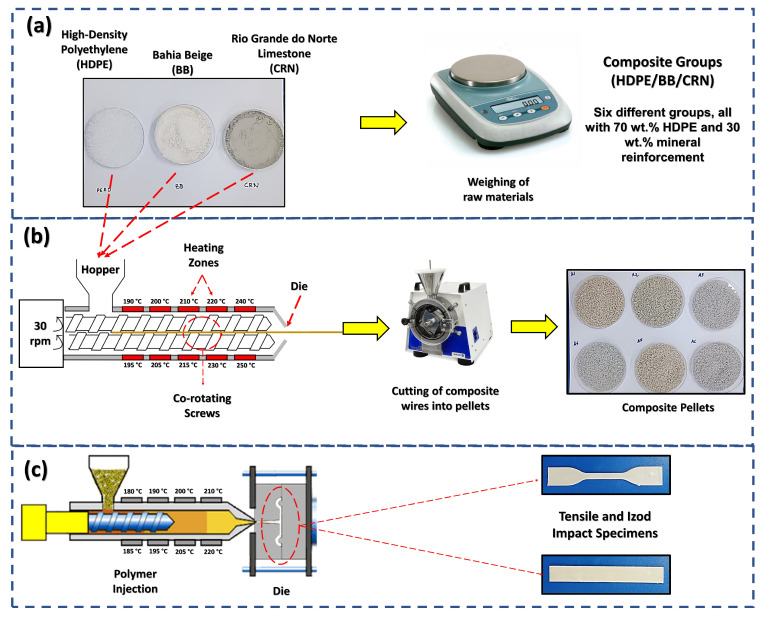
Schematic view of the processing of PEAD/BB and PEAD/CRN composites: (**a**) weighing of raw materials; (**b**) extrusion of mixtures into composite pellets; (**c**) composites’ injection into mechanical test specimens.

The composite manufacturing process is illustrated in Figure 1. Six formulations (A1 to A6) were selected, weighed, and named according to Table 1. The samples were processed using a twin-screw co-rotating extruder (Teck Tril, model DCT 20–40) with 10 temperature zones ranging from 190 to 250 °C, from the feed to the mold exit, and a rotation speed of 30 rpm. After extrusion, the materials were crushed and stored in a desiccator for subsequent preparation of test specimens for characterization.

The test specimens for tensile and Izod impact tests were prepared via injection molding using an Injection Molding Machine (Battenfeld UNILOG B2, São Paulo, Brazil). During the injection process, temperatures ranging from 180 to 220 °C were employed from the melting process to the injection nozzle. Subsequently, the impact test samples were cut using a notcher equipped with a V-notch knife, in accordance with the adopted standard, and measured separately with a digital caliper to ensure dimensional accuracy before the impact resistance tests were conducted.

### 2.3. Characterization

#### 2.3.1. Density Measurement

The composites’ density was evaluated following the technique stipulated in the ASTM D792-08 standard [48], requiring the preparation of test specimens measuring 1 cm × 1 cm. This analysis provides the material’s density (g/cm^3^), measured at room temperature (RT). The procedure was repeated five times for each material sample. Finally, the arithmetic mean of the determinations was calculated.

#### 2.3.2. Shore D Hardness

Shore D hardness tests were carried out according to the ASTM D2240-05 standard [49]. The measurements were performed using the Shore D Durometer (Type GS 702, Okaya, Japan), which provided the Shore D hardness value of the material. To ensure the accuracy of the results, the highest and lowest values obtained from each sample were excluded, and the arithmetic mean of the remaining five determinations of each sample group was calculated.

#### 2.3.3. Izod Impact Test

The Izod impact resistance test was conducted with the processed samples following the ASTM D-256 standard [50]. A universal pendulum impact testing machine (CEAST model 9050, Paraná, Brazil) was utilized for this purpose. The samples were securely positioned vertically and subjected to a 5.5-J load at the center delivered via a pendulum strike.

#### 2.3.4. Tensile Tests

For the tensile test, the test specimens were injected using an Injection Molding Machine (Battenfeld UNILOG B2, São Paulo, Brazil). Temperatures ranging from 180 °C to 220 °C were employed during the specimen fabrication, varying from the melting process to the injection nozzle. The tensile test was conducted using an Emic universal testing machine (Model DL 1000, Paraná, Brazil), equipped with a 5-kN load cell, following the ASTM D638-2003 standard [51]. All tests were carried out at RT with a relative humidity of 50% (±10%) and a deformation rate of 50 mm/min.

#### 2.3.5. Scanning Electron Microscopy (SEM)

The FEI Quanta 400 scanning electron microscope, coupled with the Bruker Nano Quantax 800 (Billerica, MA, USA) Energy Dispersive Detector (EDS) and automated Mineral Liberation Analyzer (MLA) system, was used to observe specimens coated with gold. Cryogenically fractured transversal sections of the samples were assessed, and the images were obtained at 1000× magnification.

#### 2.3.6. X-ray Diffraction (XRD)

X-ray diffraction analysis was carried out using the Bruker AXS D8 ECO X-ray diffractometer (Billerica, MA, USA) with a Cu tube, a LynxEye XE detector, and a DBO (dynamic beam optimization) system.

#### 2.3.7. X-ray Fluorescence (XRF)

The mineral residue was analyzed for its chemical composition via semi-quantitative analysis using the XRF technique in an X-ray Fluorescence Spectrometer (WDS-2), model Axios Max, Panalytical. The determination of loss on calcination was performed simultaneously using the Leco TGA-701 equipment (St. Joseph, MI, USA), with two heating ramps, one from 25–107 °C at 10 °C/min and the second ramp from 107–1000 °C at 40 °C/min. After 3 identical sequential weighings, the test was completed.

### 2.4. Statistical Analysis

StatSoft’s Statistica Academic Ultimate software was used for the statistical analysis of the results. This study used multiple linear regression to obtain mathematical models that relate the characteristics of polymeric composites based on high-density polyethylene (HDPE) to optimize mechanical performance, allowing their use in the construction industry, such as in flooring and coverings.

Variance analysis (ANOVA) was performed to evaluate such models for optimization. The study of tensile properties through the planning of experiments was carried out with three factors, one quantitative variable and two dummy variables. The quantitative factor was the MFI (melt flow index), and the dummy variables were RW (rock waste) and D (particle diameter). Information about each factor and the corresponding levels is presented in Table 2.

A fractional factorial design was used with 5 replications for the response variables density (DEN) and hardness (H), totaling 30 experiments for each variable. On the other hand, the tensile tests also followed a fractional design, but with four replicates, totaling 24 experiments for each response variable, namely the following: the Izod impact resistance J/m^2^ (IR), tensile strength (Ts), and elastic modulus (Em).

The experimental matrices included 6 experiments for each replicate, combining the minimum and maximum levels, as shown in Table 3.

Pareto charts were also obtained to determine the relation between the effects of factors and interactions with the response variables. The reference line in the graph indicates which effects are significant. In this study, the Lenth method was used to draw the reference line. Terms with effects to the right of the line represent significant parameters.

## 3. Results and Discussion

### 3.1. XRF Results

Table 4 presents the chemical composition of the two minerals used as reinforcement in the composites, Bege Bahia (BB) and Rio Grande do Norte Limestone (CRN), determined via X-ray fluorescence.

The CaO content was quite similar in both samples, with CRN containing 32.7% and BB containing 32.1%. This high CaO content was expected since both materials are types of limestone. The MgO content was slightly higher in BB (20.7%) compared to CRN (18.8%). The higher MgO content in BB may suggest a greater presence of dolomite (CaMg(CO_3_)_2_) in this sample.

XRF analysis revealed that the BB and CRN samples had similar chemical compositions, with some variations in MgO, SO_3_, K_2_O, SrO, Cl, and Na_2_O levels. These variations could have influenced the physical and chemical properties of the samples and should be taken into consideration in their industrial applications. The low concentration of rigid and oxidative elements, such as SiO_2_ and Fe_2_O_3_, is important to prevent damage to the equipment during processing.

### 3.2. XRD Results

**Figure 2:**
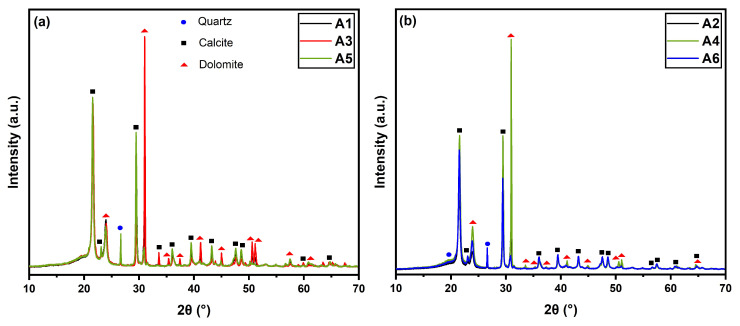
XRD patterns of HDPE composites: (**a**) A1, A3, and A5 groups; (**b**) A2, A4, and A6 groups.

The diffractograms of the HDPE/BB/CRN composites are shown below in Figure 2.

The diffractograms in Figure 2 show three distinct mineral phases for both BB-reinforced and CRN-reinforced composites. The following mineral phases were identified: calcite (JCPDS 05-0586), dolomite (JCPDS 83-1530), and quartz (JCPDS 46-1045). These phases exhibited variations only in peak intensity due to the mineral concentrations in natural rocks. The peaks indicated with arrows (around 21 and 24°) correspond to the lattice planes (110) and (200) of HDPE crystals and did not show significant variation among the different sample variations, regardless of the polymer’s MFI or the type and size of the particles [52]. The mineral phases appeared in a greater presence due to the higher crystallinity of the mineral reinforcements compared to the semicrystalline polymer matrix.

### 3.3. Physical and Mechanical Properties

The average results of the physical and mechanical properties (density, hardness, impact resistance, tensile strength, and elastic modulus) are shown in Table 5.

In Table 5, the experimentally obtained results are displayed alongside the previously obtained values for pure HDPE [39]. Regarding the density results, there were no significant changes in density values concerning particle size or the polymer’s MFI. HDPE exhibited an average density of 0.954–0.96 g/cm^3^, indicating that the density of the composites did not increase significantly and remained close to the value of pure polymer [39,53]. The samples showed good homogeneity, with density values ranging from 1.068 to 1.175 g/cm^3^, demonstrating the effectiveness of processing in dispersing the load for both BB and CRN. These results are consistent with those obtained by Chagas et al. [6], who developed recycled polypropylene (rPP) composites with additions of Bahia Beige and coconut fibers, obtaining density values between 0.829 and 1.096 g/cm^3^, for which the increase in density was minimal. Variations in density can be attributed to differences in particle compaction and roughness, as observed in sample A6, which exhibited higher values [27].

The Shore D hardness results showed good homogeneity in the load distribution within the matrix, resulting in low variation between the averages. The increase in hardness due to the addition of both BB and CRN shows that the particles increased the hardness of the composite, with the pure polymer exhibiting a hardness of 46.00 ± 2.50 Shore D. The groups with CRN reinforcement showed slightly higher values than the groups using BB as reinforcement; however, since all hardness values are within the standard deviation range, the hardness values of the groups can be considered statistically equal.

The results of the Izod impact test show that the effect of the particle size of the reinforcement agents was more pronounced. Groups A4 and A3 exhibited slightly higher impact resistance due to the smaller particle size, combined with the lower MFI of the HDPE. In this situation, regardless of whether the reinforcement was CRN or BB, these two groups showed similar values. The samples with an MFI of 25 g/10 min exhibited lower values; however, they were still close to each other, reinforcing the notion that this difference was not very significant. Pure HDPE showed the lowest impact resistance among the groups, with an average value of 2.41 ± 0.42 kJ/m^2^. This average is statistically similar to the values of groups A1 and A2, but due to the reduced MFI and increased particle size in groups A3 to A6, the performance of HDPE became inferior to that of the other composites.

The tensile strength of the composites also showed little variation among the group averages. The pure HDPE showed an average tensile strength value of 26.65 ± 1.64 MPa. The addition of particulate reinforcements, whether BB or CRN, resulted in a reduction in the tensile strength of the composites. This occurred due to discontinuity in the polymeric matrix caused by the addition of different materials, forming an interface and reducing the ability to deform plastically, thereby decreasing its ductility [54]. Groups A1 and A2, which used reinforcements with smaller particle sizes, exhibited lower values than the other groups, while group A6 also showed a low tensile strength value. The groups with a lower MFI (A3 and A4) demonstrated the best tensile strength results, and group A5 showed an intermediate result. Since the wt.% of the composite was not altered, the changes were not very significant, but high additions of the ceramic load can considerably reduce the tensile strength of HDPE. For example, Cardoso et al. [39] added fractions of 40, 50, and 60 wt.% of Al_2_O_3_ to HDPE and observed a reduction from 14.44 MPa for the group with 40 wt.% of Al_2_O_3_ to 12.38 MPa for the group with 60 wt.% of ceramic reinforcement. Regarding the tensile modulus, groups A1 and A2 showed the highest average values, likely due to greater deformation. Group A6 exhibited the lowest value among all groups, highlighting a relatively significant difference, with group A1 having a modulus of 335.50 MPa, while group A6 has a modulus of 277.50 MPa. It is possible that the larger CRN particles did not achieve effective homogenization, reducing the composite’s tensile modulus.

**Figure 3:**
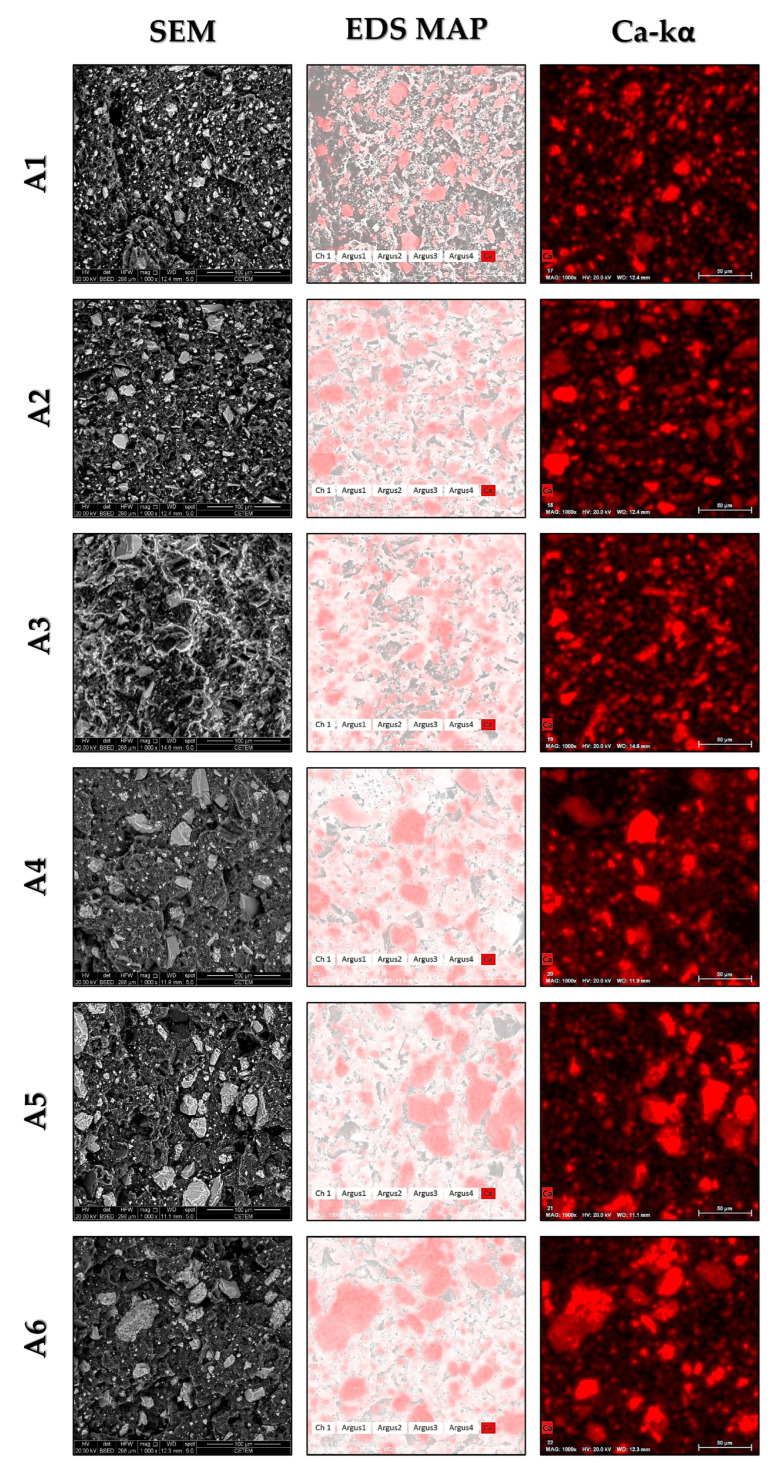
Micrographs of fracture surfaces of HDPE/BB/CRN composites. Left: SEM images obtained using a secondary electron detector. Center: EDS map overlay highlighting calcium distribution on the sample. Right: calcium distribution map.

The morphology of the fractured surfaces of the composites after the Izod impact test is illustrated in Figure 3. On the left side of Figure 3, the micrographs of the composites reveal a highly porous morphology in samples A1 and A2. The higher MFI of these two groups appears not to have fully filled the spaces during composite processing for either BB or CRN. This resulted in low interfacial adhesion, leading to particle pull-out during the impact test, as indicated by the high quantity of visible pores. The reduction in MFI, coupled with an increase in particle size, resulted in a less porous surface, as observed in groups A3 and A4; however, sample A4 still exhibited characteristics of low interfacial adhesion, evidenced by voids at the matrix/reinforcement interface. Samples A5 and A6 appear to have undergone densification, as the surface showed fewer pores. However, in sample A5, BB agglomeration occurred, resulting in larger holes due to particle pull-out during the impact test. Sample A6 also showed agglomerates, but the pores were smaller.

In the center and right sections of Figure 3, EDS maps are presented, showing the overlay of calcium on the sample and its isolated distribution. Samples A1 and A2, fabricated with the smallest particle size, exhibited smaller and well-distributed particles across the surface, indicating good particle dispersion. In contrast, in samples A5 and A6, where only the particle size was altered compared to these samples, regions with a higher predominance of particles can be observed, indicating a lower dispersion efficiency. Samples A3 and A4, produced with the same particles as A5 and A6 but with an increased viscosity of the polymer matrix, showed a reduction in the average agglomerate formation. There is a greater tendency for agglomeration with lower viscosity, as the viscous phase provides the necessary forces to disperse the particles [55,56].

### 3.4. Statistical Analysis of Physical and Mechanical Properties of Composites

**Figure 4:**
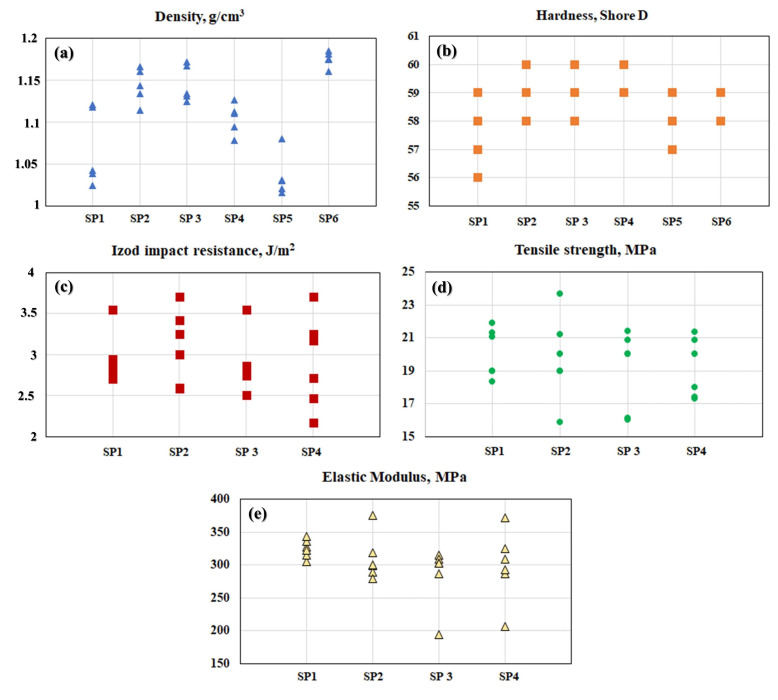
Values of response variables for the following: (**a**) density; (**b**) hardness of the specimens (SP1–SP6); (**c**) Izod impact resistance; (**d**) tensile strength; and (**e**) elastic modulus of the specimens (SP1–SP4).

The responses measured for the physical and mechanical properties are presented in Figure 4.

Table 6 presents the statistical results of the regression performed for each response variable. The adjusted R-Sq (adj) values for the regression models of the DEN, H, IR, Ts, and Em responses are 95.78%, 95.81%, 93.76%, 93.96%, and 92.78%, respectively. These coefficients present levels above 90%, which implies that the models have good predictability. The F Statistics, the result of the ANOVA in regression, allows for the testing of the joint effect of the factors on the response variable; that is, it serves to verify whether at least one of the factors explains the variation in the response variable. A significance level of 0.05 is considered; if Significance F ≤ 0.05, the regression is significant, but if it is >0.05, the regression is not significant. After analyzing the F Statistics, presented in Table 6, it is possible to suggest that the five response variables can be explained by the factors used in the study.

ANOVA tables (Table 7, Table 8, Table 9, Table 10 and Table 11) were used to detect the factors and their interactions that significantly influence composite properties. The *p*-values for the factors (MFI, RW, and D) define the influence on the response variables (Den, H, IR, Ts, and Em). As the level of confidence was considered at 95%, then, if the *p*-value of the factor or the interaction is lower or equal to the risk degree (0.05 or 5%), there is a significant correlation between the response variables and the factor, while *p*-values higher than 0.05 show the absence of a correlation.

Using the coefficients shown in Table 7, Table 8, Table 9, Table 10 and Table 11, the regression models for each response were obtained and represented in Equations (1)–(5). Negative values in the regression models indicate that the behavior of the factor or the interaction evaluated is inversely proportional to the response variable.

(1) DEN=0.043·MFI−0.1764·RW+1.188·D+0.010·MFI·RW−0.049·MFI·D+0.065·RW·D±0.026

(2) H=2.296·MFI+59.47·D−2.363·MFI·D±0.866

(3) IR=0.1046·MFI·1.046·RW+3.044·D−0.041·MFI·RW−0.107·MFI·D±0.252

(4) Ts=0.79·FMI+21.319·D−0.808·MFI·D±1.401

(5) Em=13.42·MFI+285.972·D−12.559·MFI·D±40.52

**Figure 5:**
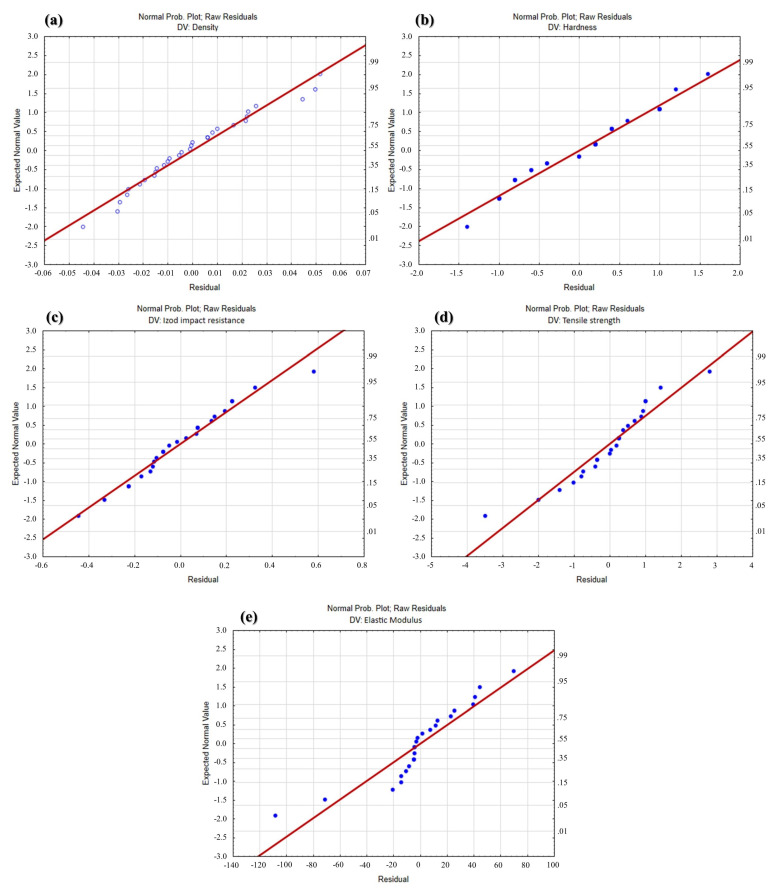
Normal distribution of residual regression models: (**a**) density; (**b**) hardness; (**c**) impact resistance; (**d**) tensile strength; and (**e**) elastic modulus.

Residual analysis was performed to check for the assumptions of the ANOVA and validate the regression models. Figure 5 shows the normal distribution graphs of the residuals obtained from the regression models. A well-adjusted model must be able to represent all the systematic information contained in the data. The residues left by it must represent only the random part, that is, the noise embedded in the measurements. Therefore, the behavior of the residuals in this graph should be very close to that of a random sample drawn from a zero-mean normal distribution.

Analyzing Figure 5 reveals that this effect is true for all models, demonstrating the good quality of the regression models.

The experimental results obtained resulted in five regression models with a good predictive capacity (adjusted R^2^ > 0.92) and low variability. The first- and second-degree interactions considered in the study were satisfactory for all response variables.

**Figure 6:**
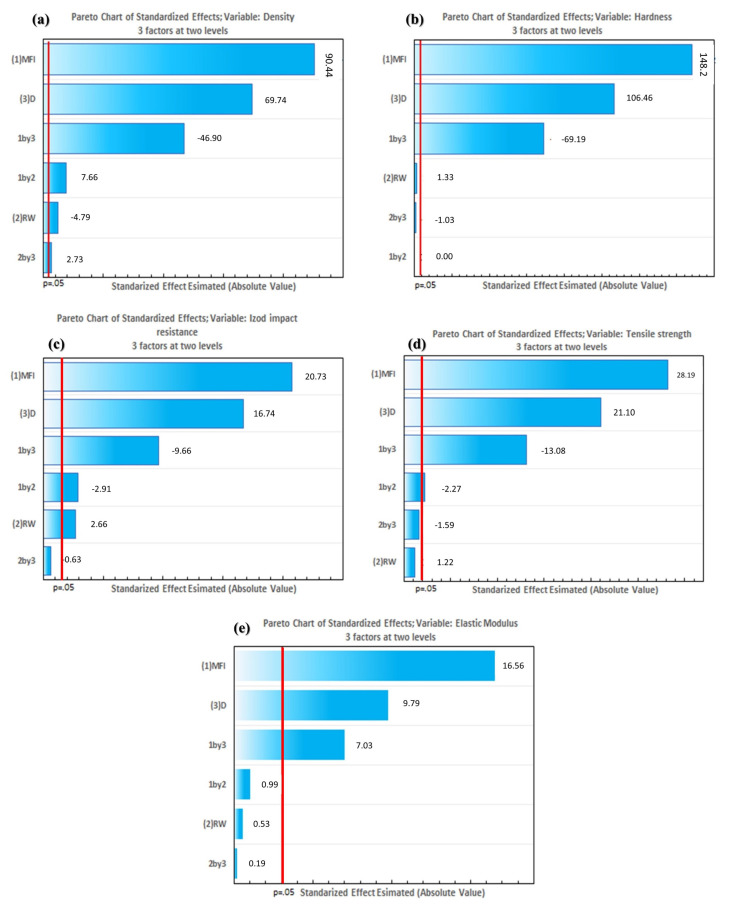
Pareto chart: (**a**) density; (**b**) hardness; (**c**) impact resistance; (**d**) tensile strength; and (**e**) elastic modulus.

As shown in the ANOVA results, the MFI (melt flow index) and D (particle diameter) factors were significant for all response variables. The Pareto charts (Figure 6) demonstrated that the MFI was the most significant factor in all regression models, followed by factor D. As shown in all regression models, the MFI*RW interaction was significant for all response variables, resulting in the third most statistically significant term, according to the Pareto charts.

The RW factor (rock waste) and its interactions with other factors were significant only for density and Izod impact resistance, showing low statistical significance in the Pareto charts, present in Figure 6, compared to the other terms of the regression model.

The Pareto charts analyzed the effects on different variables (density, hardness, Izod impact strength, tensile strength, and the elastic modulus) for three factors at two levels. The negative values observed in the charts indicate that an increase in the level of these factors leads to a decrease in the response variable. When considering the density variable in Figure 6a, it was observed that the interaction between factors 1 (MFI) and 3 (ID) presented a significant negative effect (−46.90), indicating that the simultaneous increase in the levels of these factors reduces the material’s density. The interaction between factors 2 (RW) and 1 (MFI) also resulted in a decrease in density, although with a lower magnitude (−4.79). Regarding the hardness variable represented in Figure 6b, the interaction between factors 1 (MFI) and 3 (ID) had a notable negative effect (−69.19), demonstrating that hardness decreases when both levels of these factors increase. The interaction between factors 2 (RW) and 3 (ID) also presented a small negative effect (−1.03). For the Izod impact strength variable (Figure 6c), the interaction between factors 1 (MFI) and 3 (ID) again presented a negative effect (−9.66), indicating that impact strength decreases with the increase in these levels. The interaction between factors 1 (MFI) and 2 (RW) also leads to a reduction in impact strength (−2.91). In the case of tensile strength (Figure 6d), the interaction between factors 1 (MFI) and 3 (ID) showed a significant negative effect (−13.08), reducing the material’s tensile strength. The interaction between factors 1 (MFI) and 2 (RW) also resulted in a decrease in tensile strength (−2.27). Finally, for the elastic modulus in Figure 6e, the interaction between factors 1 (MFI) and 3 (ID) had a negative effect (−7.03), indicating that the elastic modulus decreases with the increase in these levels. The interaction between factors 1 (MFI) and 2 (RW) also leads to a reduction in the elastic modulus (−0.99).

The negative values in the Pareto charts indicate that the interactions between the corresponding factors lead to a decrease in the analyzed response variables. This suggests that increasing the levels of these factors is not beneficial for the specific properties of the studied material, negatively impacting the density, hardness, Izod impact strength, tensile strength, and elastic modulus.

## 4. Conclusions

In this study, the influence of the HDPE melt flow index (MFI) and particle size of two different mineral reinforcements (Bahia Beige and Rio Grande do Norte Limestone) was evaluated through physical, chemical, and microstructural characterizations, supported by a statistical analysis. The chemical analysis revealed the composition of both mineral reinforcements, showing a high content of CaO, followed by MgO and other minor oxides. The variation in composition between the two minerals was minimal, as confirmed via an XRD analysis, which identified dolomite, calcite, and quartz phases in both mineral-reinforced composites.

Density testing indicated higher values for composites with MFI = 25 g/10 min and a larger CRN particle size (20 < D < 44 μm), suggesting greater densification and compaction during processing. Composite hardness exhibited minimal variation, although it was influenced by the particle size. Izod impact testing showed that composites with lower MFIs demonstrated higher impact resistance; the A4 sample group presented an impact resistance of 3.62 kJ/m^2^. Tensile testing results indicated superior performance for composites with lower MFIs and larger particle sizes; the A3 group showed a tensile strength equal to 21.19 MPa, the greatest value reached. Particle agglomeration was observed across all sample groups due to processing, as evidenced in the SEM/EDS analyses, contributing to a porous surface indicative of low interfacial adhesion.

Statistical analysis demonstrated high reliability, with high R^2^ values and low F-test significances, indicating statistically similar means among the studied groups. Ultimately, the study found that employing Bahia Beige and Rio Grande do Norte limestone, and varying HDPE MFIs, did not significantly influence the physical and mechanical properties of the composites.

Based on the results of the study, the authors propose the following composite structure for further application:

- Filler type: Both Bahia Beige and Rio Grande do Norte Limestone are recommended as effective mineral reinforcements due to their similar chemical compositions and phase structures.
- Filler particle size: A larger particle size is recommended, as it was found to contribute to superior tensile performance and higher density values.
- MFI of polyethylene: A lower MFI of HDPE is proposed, as it demonstrated higher impact resistance and better tensile performance.

In summary, the optimal composite composition for future applications includes either Bahia Beige or Rio Grande do Norte Limestone with larger particle sizes and HDPE with a lower MFI. This combination is expected to enhance the mechanical properties and overall performance of the composite material. It is recommended to utilize these composites as flooring and coatings in the civil industry. Further analysis is advisable.

## Figures and Tables

**Table 1:** Details of the formulations of the processed sample groups.

Sample	Composite Configuration	Matrix/Mineral (wt.%)	MFI HDPE (g/10 min)	Particle Size Mineral Filler
A1	HDPE/BB	70/30	25	D < 20 μm
A2	HDPE/CRN	70/30	25	D < 20 μm
A3	HDPE/BB	70/30	7	20 < D < 44 μm
A4	HDPE/CRN	70/30	7	20 < D < 44 μm
A5	HDPE/BB	70/30	25	20 < D < 44 μm
A6	HDPE/CRN	70/30	25	20 < D < 44 μm

HDPE—high-density polyethylene; BB—Bahia Beige; CRN—Rio Grande do Norte Limestone (*Calcário do Rio Grande do Norte*).

**Table 2:** Factors used in the statistical analyses.

Factors	Level Values	
** **	**Minimum**	**Maximum**
MFI, g/10 min	7	25
RW	0 (Bahia Beige marble waste)	1 (limestone waste from Rio Grande do Norte)
D	0 (D_1_ < 20 mm)	1 (20 mm < D_2_ < 44 mm)

**Table 3:** Experimental matrix used for each replicate.

Experiment	MFI	RW	D
1	7	0	1
2	7	1	1
3	25	0	0
4	25	0	1
5	25	1	0
6	25	1	1

**Table 4:** Chemical composition of CRN and BB powders analyzed via XRF.

Sample	CaO	MgO	SiO_2_	Al_2_O_3_	SO_3_	K_2_O	Fe_2_O_3_	SrO	Cl	P_2_O_5_	Na_2_O	LOI ^1^
CRN	32.7	18.8	1.1	0.37	0.04	0.06	0.16	0.03	0.06	0.09	0.39	46.2
BB	32.1	20.7	1.0	0.37	ND	ND	0.16	ND	ND	0.1	0.09	45.5

^1^ LOI = loss on ignition.

**Table 5:** Summary results of the physical and mechanical properties of composites.

Sample	Density (g/cm^3^)	Hardness (Shore D)	Impact Resistance (kJ/m^2^)	Tensile Strength (MPa)	Elastic Modulus (MPa)
HDPE [39]	0.96	46.00 ± 2.50	2.41 ± 0.42	26.65 ± 1.64	-
A1	1.07 ± 0.05	57.40 ± 1.14	2.61 ± 0.33	19.75 ± 0.50	335.50 ± 26.85
A2	1.14 ± 0.02	59.00 ± 1.00	2.64 ± 0.14	18.00 ± 1.41	313.25 ± 15.88
A3	1.14 ± 0.02	59.10 ± 1.01	3.02 ± 0.26	21.19 ± 0.49	292.00 ± 8.72
A4	1.10 ± 0.02	59.80 ± 0.45	3.62 ± 0.09	20.40 ± 1.12	302.25 ± 77.99
A5	1.03 ± 0.03	57.80 ± 0.84	2.97 ± 0.14	20.87 ± 2.59	307.50 ± 11.82
A6	1.17 ± 0.01	58.60 ± 0.55	2.84 ± 0.40	16.90 ± 1.13	277.50 ± 50.81

**Table 6:** Statistical results of regression models for each response variable.

Model of	R-sq, %	R-sq (adj), %	Significance F
Density (DEN)	99.95%	95.78%	1.70 × 10^−37^
Hardness (H)	99.98%	95.81%	3.15 × 10^−42^
Izod impact resistance J/m^2^ (IR)	99.46%	93.76%	1.55 × 10^−18^
Tensile strength (Ts)	99.62%	93.96%	8.65 × 10^−20^
Elastic modulus (Em)	98.69%	92.78%	2.90 × 10^−15^

**Table 7:** ANOVA of factorial design for density (DEN).

Term	Coefficients	St. Error	Stat t	*p*-Value
MFI	0.0427	0.0005	90.4450	6.34 × 10^−32^
RW	−0.1764	0.0368	−4.7905	7.08 × 10^−5^
D	1.1882	0.0170	69.7388	3.18 × 10^−29^
MFI-RW	0.0101	0.0013	7.6612	6.73 × 10^−8^
MFI-D	−0.0488	0.0010	−46.9029	4.04 × 10^−25^
RW-D	0.0646	0.0236	2.7343	0.0116

MQ: means squared; SQ: sum of squares; DF: sum of squares.

**Table 8:** ANOVA of factorial design for hardness (H).

Term	Coefficients	St. Error	Stat t	*p*-Value
MFI	2.2960	0.0155	148.2062	4.61 × 10^−37^
RW	1.6000	1.2072	1.3253	0.1975
D	59.4667	0.5586	106.4561	1.28 × 10^−33^
MFI-RW	0.0000	0.0430	0.0000	1.0000
MFI-D	−2.3627	0.0341	−69.1938	3.89 × 10^−29^
RW-D	−0.8000	0.7746	−1.0328	0.3120

MQ: means squared; SQ: sum of squares; DF: sum of squares.

**Table 9:** ANOVA of factorial design for Izod impact resistance (IR).

Term	Coefficients	St. Error	Stat t	*p*-Value
MFI	0.1046	0.0050	20.7329	5.17 × 10^−14^
RW	1.0458	0.3931	2.6601	0.0159
D	3.0444	0.1819	16.7355	2.04 × 10^−12^
MFI-RW	−0.0408	0.0140	−2.9137	0.0093
MFI-D	−0.1074	0.0111	−9.6563	1.53 × 10^−8^
RW-D	−0.1600	0.2523	−0.6343	0.5339

MQ: means squared; SQ: sum of squares; DF: sum of squares.

**Table 10:** ANOVA of factorial design for tensile strength (Ts).

Term	Coefficients	St. Error	Stat t	*p*-Value
MFI	0.7900	0.0280	28.1902	2.40 × 10^−16^
RW	2.6674	2.1838	1.2215	0.2377
D	21.3187	1.0105	21.0976	3.82 × 10^−14^
MFI-RW	−0.1767	0.0778	−2.2699	0.0357
MFI-D	−0.8077	0.0618	−13.0773	1.25 × 10^−10^
RW-D	−2.2222	1.4012	−1.5859	0.1302

MQ: means squared; SQ: sum of squares; DF: sum of squares.

**Table 11:** ANOVA of factorial design for the elastic modulus (Em).

Term	Coeficients	St. Error	Stat t	*p*-Value
MFI	13.4200	0.8105	16.5578	2.44 × 10^−12^
RW	33.6528	63.1589	0.5328	0.6007
D	285.9722	29.2245	9.7854	1.25 × 10^−8^
MFI-RW	−2.2361	2.2514	−0.9932	0.3338
MFI-D	−12.5589	1.7864	−7.0303	1.47 × 10^−6^
RW-D	−7.7500	40.5247	−0.1912	0.8505

MQ: means squared; SQ: sum of squares; DF: sum of squares.

## Data Availability

The original contributions presented in the study are included in the article, further inquiries can be directed to the corresponding authors.
